# Role of MWCNTs Loading in Designing Self-Sensing and Self-Heating Structural Elements

**DOI:** 10.3390/nano13030495

**Published:** 2023-01-26

**Authors:** Liberata Guadagno, Raffaele Longo, Francesca Aliberti, Patrizia Lamberti, Vincenzo Tucci, Roberto Pantani, Giovanni Spinelli, Michelina Catauro, Luigi Vertuccio

**Affiliations:** 1 Department of Industrial Engineering, University of Salerno, Via Giovanni Paolo II, 132, 84084 Fisciano, Italy; 2 Department of Information and Electrical Engineering and Applied Mathematics University of Salerno, Via Giovanni Paolo II, 132, 84084 Fisciano, Italy; 3 Faculty of Transport Sciences and Technologies University of Study “Giustino Fortunato”, Viale Raffaele Delcogliano 12, 82100 Benevento, Italy; 4 Open Laboratory on Experimental Micro and Nano Mechanics, Institute of Mechanics, Bulgarian Academy of Sciences, Acad. Georgi Bonchev Street, Block 4, 1113 Sofia, Bulgaria; 5 Department of Engineering, University of Campania “Luigi Vanvitelli”, Via Roma 29, 81031 Aversa, Italy

**Keywords:** electrical properties, self-heating, self-sensing, thermosetting resin, carbon nanotubes

## Abstract

This work proposes nanocomposites with carbon nanotubes characterized by self-sensing and self-heating properties. Recently, a growing interest in these two properties has been found in many industrial sectors, especially in the aerospace and automotive fields. While the self-sensing function allows diagnosing the presence of micro-damage in the material thanks to the detection of residual resistance, the self-heating function is exploited to properly tune the heating performance in terms of the heating rate and final temperature values. An electrical percolation value of around 0.5% by weight of carbon nanotubes was found by electrical characterization. The AC conductivity of the nanocomposites, in the range of 100 Hz to 1 MHz, evidences that beyond a CNTs amount of 0.5% wt/wt, they are characterized by a purely resistive behavior. The self-sensing analysis displayed a gauge factor value of 4.1. The solid thermal stability up to 300 °C makes the material suitable as a heating element at high temperatures. SEM investigations and temperature maps evidence a good dispersion of the conductive filler in the epoxy matrix and, consequently, good isotropy in heat distribution. As regards the trend of electrical resistance by varying the temperature, the electro-thermal investigation has shown the presence of both Positive Temperature Coefficient (PTC) and Negative Temperature Coefficient (NTC) behaviors with a predominance of NTC as soon as the temperature becomes closer to the glass transition temperature of the epoxy resin.

## 1. Introduction

Research for new materials plays a crucial role in developing new technologies to be applied in different sectors, from medicine, transport, and information technology, up to the civil engineering components. An accurate study of the strict relationship between a material’s structure and properties is the starting point for designing new and more efficient systems. This understanding allows designing hybrid materials to satisfy new needs related to the materials’ performance. Polymers are the most used materials thanks to their low cost, ease of processing, reproducibility, and stability [1]. The combination of the latter with a wide range of fillers, having one or more dimensions in the nanometer range, has led to the development of polymeric nanocomposites with improved properties and new potential applications [2,3,4,5,6,7,8,9,10]. More specifically, the high aspect ratio, mechanical strength, and exceptional electrical properties make carbonaceous nanofillers such as graphene and carbon nanotubes (CNTs) the best filler for this scope [11,12,13,14,15,16,17,18]. In addition, due to their nanometric dimensions, these nanofillers have a large specific surface that maximizes the contact area between filler particles and the polymeric matrix, such that the transfer of mechanical stress between them, indeed the mechanical properties of the whole material, are improved [19,20,21,22,23,24,25,26]. Regarding electrical conductivity, carbon nanofillers with a high aspect ratio favor the formation of conductive pathways, which is essential for obtaining a low percolation threshold [27,28,29,30,31,32,33], while as far as the thermal conductivity of the composite is concerned, an increase in the size of the filler improves the heat transfer at the nanoparticle/nanoparticle–polymer interface due to the increase in the value of the interfacial thermal conductance (G) [34,35,36].

The properties described up to now can be exploited for the realization of “smart” materials able to react appropriately (output) as a response to an external stimulus (input) on the type: physical, mechanical, electrical, chemical, thermal, optical, etc. Sensor-integrated systems, such as sensing changes in strain, pressure, and temperature, can be easily built with a lightweight structure and without additional equipment, satisfying exactly the requirements of industrial applications in the areas of civil, mechanical, and aerospace engineering. Likewise, smart nanomaterial heaters with high mechanical performance are considered for electrothermal performance (e.g., antifreeze, rapid cleaning of crude oil, and mass heating engineering) [37,38,39,40]. However, the current scientific literature is centered mostly only on smart composite materials with excellent performance as structural components and heating and sensing elements separately [41,42]. In a scenario where the above-mentioned results are limited to a single function (sensing or heating), the present study aims to produce a CNT-reinforced composite as a smart system that can be applied both as a strain sensor and a heating element, provided that the concentration of the filler is suitably modulated.

## 2. Materials and Methods

The epoxy resin studied in this work consists of a 3,4-epoxycyclohexylmethyl-3’,4’-epoxycyclohexane carboxylate (ECC) with a methyl hexahydrophthalic anhydride (MHHPA) as a curing agent (see Figure S1 of Supplementary Materials). Carbon nanotubes are characterized by an outer mean diameter ranging from 10 to 15 nm, a length ranging from 0.1 to 10 μm, and several walls varying between 5 and 15 [29] (see Supplementary Materials). Furthermore, the carbonaceous filler is characterized by the presence of polar groups on the walls (see Figures S2 and S3 of Supplementary Materials), and by an intensity ratio of the D and G bands (I_D_/I_G_) equal to 1.38 (see Figure S4 of Supplementary Materials).

Epoxy precursor, hardener, and carbon nanotubes are mixed following the procedure described in Spinelli et al. [43] to produce samples with different concentrations. Table 1 summarizes the several methods and the experimental techniques employed for this research work. These procedures have already been adopted in previous papers [43,44,45,46,47]. Regarding the electro-mechanical characterizations, because the specimen resistance consists of several kΩ, a two-probe measurement has been adopted, considering the contact resistance negligible [48,49].

**Figure 1:**
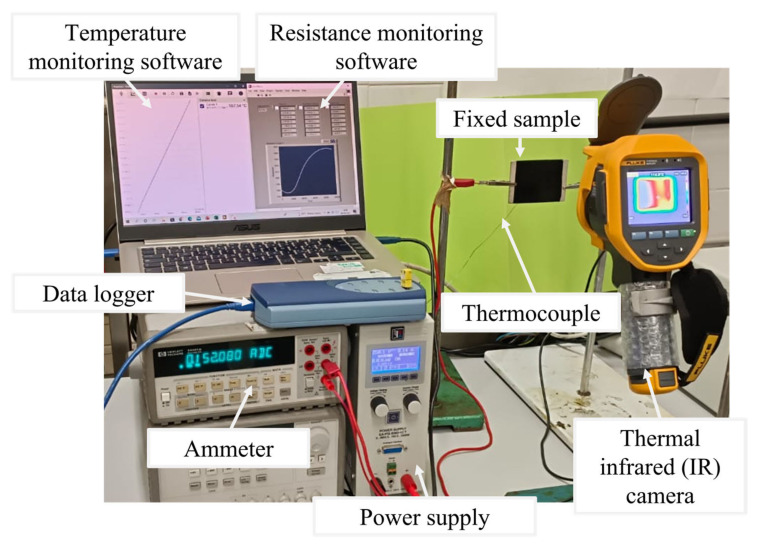
Equipment picture of the Joule heating test.

To perform the Joule heating test, electrical contacts realized on the short sides of the sample with silver paint (RS 196-3600, RS PRO, Corby, UK) were connected to the power supply EA-PSI 8360-10T (Elektro-Automatik, 0–360 V, 0–10 A, 1000 W max) and the HP34401 A ammeter (min current 0.1 µA) according to the two-probe configuration (see Figure 1). Applying determined voltage values between 70V and 200V to the sample, the local temperature evolution over time was measured by a thermocouple positioned at the center of the sample surface and recorded by a data acquisition board (Data Logger TC-08 supplied by Pico Technology) interfaced with the PicoLog dedicated software (version 6.2.5). 

The employed T-type thermocouple with a diameter of about 100 μm and negligible thermal inertia was supplied by Omega Engineering Ltd. The temperature distribution on the surface of the sample was monitored by a thermal infrared (IR) camera (Fluke Ti401 Pro Thermal Imager, Everett, WA, USA) in a fixed position in front of the sample (see Figure 1). Each heating test was performed in direct current (DC) mode, and the applied voltage value was kept constant for 3600 s to reach a steady-state condition. The electrical current measured by the ammeter was recorded by a data acquisition board (National Instruments GPIB-USB-HS, Austin, Texas, USA) connected to a specific LabView program. Because the electrical characteristic reveals a purely ohmic behavior of the nanocomposite material, once the voltage is fixed, for each electrical current recorded value the LabView program calculates the resistance value by tracing its trend over time during the heating test. 

Regarding boundary conditions, the sample was maintained in a fixed position in static air at an ambient temperature of about 25 °C. Preliminary tests showed that the temperature values on both surfaces of the sample present differences of less than 2 °C. For this reason, with a reasonable estimate, it has been considered to neglect the difference in temperature values along the sample’s normal section and evaluate the temperature profile by placing a single thermocouple on the surface.

## 3. Results and Discussion

### 3.1. Electrical, Morphological and Rheological Properties

#### 3.1.1. DC Measurements

Electrical characterization was necessary in order to choose the amount of filler most suitable for imparting the desired functionality. Usually, the behavior of composites based on conductive fillers and insulating matrices is explained by the theory of electrical percolation. Including the electrically conductive filler into a polymer causes an increase in electrical conductivity, resulting in a transition from a non-conductive material to an electrically conductive one. In the proximity of a critical percentage of filler, the material undergoes a net increase in electrical conductivity of several orders of magnitude. This critical content is indicated as the electrical percolation threshold, i.e., the value beyond which the matrix has a stable electrically conductive network, often shown on a logarithmic scale as a saturation plateau. More specifically, the percolation theory describes the dependence of conductivity, σ, on the amount of the conductive filler through the scaling law:

(1) $$\sigma =\sigma_{0}{\left(x-x_{c}\right)}^{t}$$

where *σ*_0_ is a fitting parameter, *t* is an exponent depending on the system dimensionality, *x* is the filler amount (i.e., wt%), and *x_c_* is the percolation threshold filler amount [50,51,52,53,54,55]. By varying polymeric matrices, CNT type, synthesis and treatment method, etc., it is possible to observe variations in the values of the electrical parameters [54]. In any case, the conductive networks in the composite are obtained for concentration values of the filler above the percolation threshold.

**Figure 2:**
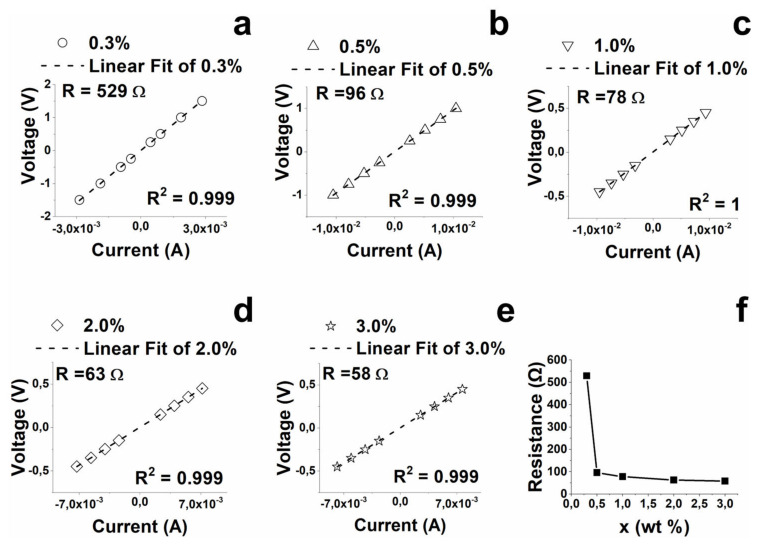
Results of the measurements voltage–current for filled systems: (**a**) 0.3%, (**b**) 0.5%, (**c**) 1.0%, (**d**) 2.0%, (**e**) 3.0%, and (**f**) relationship between electrical resistance and CNTs concentration.

The results of the voltage–current measurements are shown in Figure 2, where the electrical resistance has been obtained from the evaluation of the slope of the linear fitting of experimental data. As expected, the resistance value decreases with increasing concentrations of carbon nanotubes.

**Figure 3:**
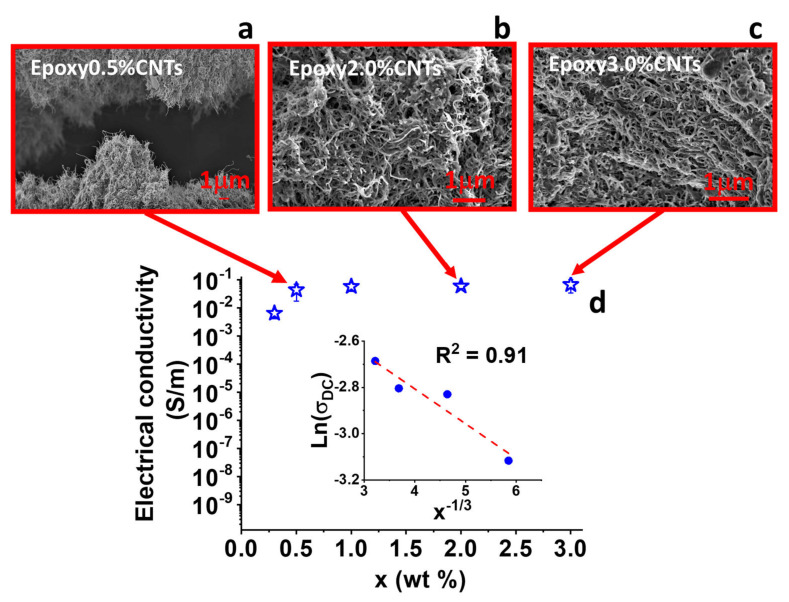
SEM images of epoxy systems filled with (**a**) 0.5, (**b**) 2.0, and (**c**) 3.0% by weight; (**d**) electrical conductivity behavior of the CNTs system. Inset: the relationship between the electrical conductivity, in logarithmic scale, and x^−1/3^.

The results of the electrical conductivity are shown in Figure 3. The electrical conductivity, σ, has been calculated using the following equation:

(2) $$\sigma =\frac{L}{A*R}$$

where *A* is the cross-sectional area of the specimen, *L* is the sample thickness, and *R* is the evaluated electrical resistance.

In Figure 3d, the electrical conductivity vs. the amount of filler is reported. According to the scaling law mentioned above, the conductivity depends on the concentration of the filler. The electrical behavior shows the typical increase in conductivity predicted by the percolation theory up to a maximum value of 6.8 × 10^−2^ S/m obtained for a filler concentration of 3% by weight. The electrical percolation threshold is attested for a value x_c_ < 0.5% by weight.

Furthermore, as already observed for composites reinforced with filler of one-dimensional type such as CNTs, if the filler content in the composite is well distributed, the macroscopic DC conductivity, above the percolation threshold, can be ascribed to tunnel conduction with a single tunnel junction because the following dependence holds:

(3) $$ln\left(\sigma_{DC}\right)\propto \;x^{-1/3}$$

The linear relationship between the electrical conductivity, in logarithmic scale and *x*^−1/3^ (see the inset of Figure 3d), confirms that the main electrical transport mechanism in this composite is ascribed to the electron tunneling phenomenon. [56]. The electrical current is limited by potential barriers between conductive fillers covered by a thin insulating layer of resin. The good distribution of the filler in the matrix is further testified by the SEM images shown in Figure 3a–c, which depict the filled samples with 0.5%, 2%, and 3%, respectively. The arrangement of the nanofiller in the matrix is very clear thanks to the previous etching treatment, which allowed for the removal of the epoxy resin, leaving stripped or partially covered walls of carbon nanotubes.

#### 3.1.2. AC Measurements

**Figure 4:**
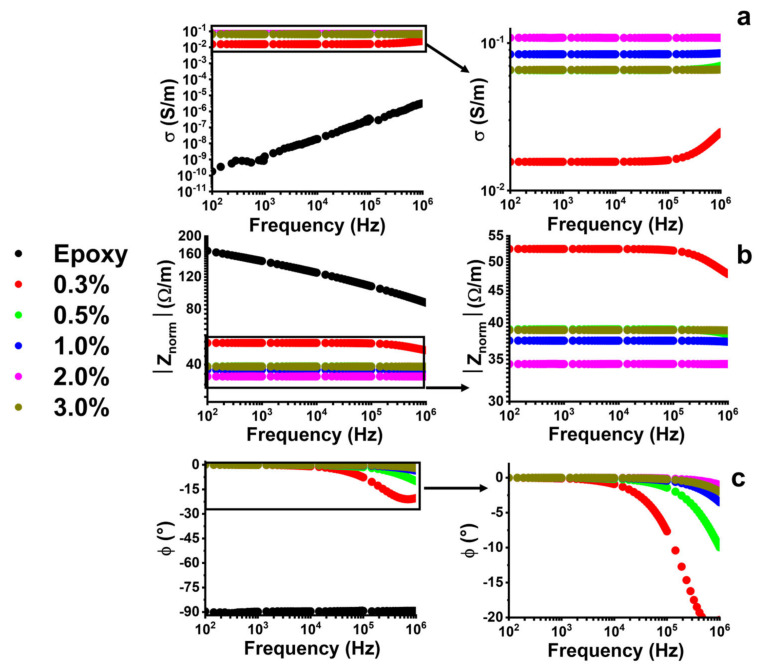
(**a**) AC electrical conductivity, (**b**) modulus, and (**c**) phase impedance of composite materials, evaluated in the frequency range of 100 Hz–1 MHz.

To explore the electromagnetic (EM) properties of multi-phase systems, experimental investigations of the AC electrical properties are required, because they play an important role in the characterization of the nanocomposites. Typically, the tool classically adopted for this aim is impedance spectroscopy, based on the evaluation of the electrical impedance as a function of the frequency of the sinusoidal stimulus. In the case of investigation on composite materials filled with conducting inclusions, it allows for observation of the insulator-to-conductor transition and of the critical frequency, f_c_, for which the composite changes from a frequency-independent behavior to a frequency-dependent one [56]. The phenomenon of electrical conduction in AC mode, in insulating materials and therefore in the unfilled resin, is dominated by dielectric losses because the transport of charge inside the material can be considered negligible. The increase in AC conductivity that is found for insulating materials with the frequency increase is due to the phenomena just mentioned. The dielectric losses are associated with the amount of energy, supplied by the application of the field, dissipated by friction between the molecules, in the form of heat. The phenomenon of continuous orientation to which the molecules are forced causes the typical increase in AC conductivity as the frequency increases [57]. The AC conductivity of the nanocomposites evaluated in the frequency range from 100 Hz up to 1 MHz is shown in Figure 4.

In the frequency range of 100 Hz–1 MHz, the epoxy resin presents an evident frequency-dependent behavior where a progressive increase in the conductivity with increasing frequency is observed. For an amount of the filler next to the EPT, the conductivity remains almost constant, with a slight increase only at high frequency in contrast to the frequency-independent conductivity, in the range of 100 Hz–1 MHz exhibited by the composite reinforced with higher filler concentrations. Such a phenomenon is typical for insulator–conductor systems: at low frequency (near to DC condition) the conductivity is independent of the frequency up to a critical value (f_c_), above which a frequency-dependent behavior is observed. In fact, for frequency values > f_c_, the AC conductivity increases. In particular, the f_c_ value is between 10^5^ Hz and 10^6^ Hz for the system with carbon nanotubes (wt% = 0.3%). After the 0.5% concentration, the electrical conductivity results independent of the frequency. Figure 4 shows the plots of the values of the normalized impedance (|Z_norm_| = |Z| × d/A in Ωm^−1^, where A is the area of the electrode and d is the thickness of the sample) and relative phase angle (expressed in degrees) as a function of the frequency. Usually for the composites with filler concentration below the EPT, the |Z_norm_| is proportional to 1/frequency (in log–log scale) and ϕ ≈ −90°, as for insulating material, is close to the trend of the neat resin. Instead, for the composite above the percolation threshold value, both the modulus and phase present a constant value until a critical value, f_c_, after which it decreases. This means that for concentration higher than the EPT, at low frequency, the resistive effects dominate the impedance, whereas for a higher frequency |Z_norm_| is mainly affected by the capacitive behavior of the material, reaching values lower than the resistance exhibited by the nanocomposite. From a closer view of Figure 4, the frequency-dependent behavior becomes less and less pronounced as the percentage of filler increases, resulting in a shift of f_c_ to higher values and indicating the transition of the material, as described above [58,59]. In fact, f_c_ is related to the tunneling phenomenon that is affected by the energy barrier and the tunneling distance. These two parameters depend on the frequency of the applied electric field and decrease for frequency values greater than the critical value (f_c_) with consequent modification of the electrical performance of the composite [56]. In light of the last considerations, the 0.5% threshold can be considered the value beyond which the composite is characterized by a purely resistive behavior in the whole range of frequencies investigated.

Downstream of the electrical characterization, a concentration of 0.5% and 3.0% by weight was chosen to evaluate the self-sensing and self-heating response, respectively. The choice of 0.5% by weight to evaluate the sensing properties was made based on previous studies [60,61] in which it is stated that a material highly sensitive to deformation can be obtained when the loaded MWCNT approaches the percolation threshold, whereas a concentration of 3.0% by weight was chosen to evaluate the heating properties to minimize the applied voltage values, as the voltage is a very important technological parameter if we consider the limited generators available in the various fields of application.

#### 3.1.3. Rheological Properties

**Figure 5:**
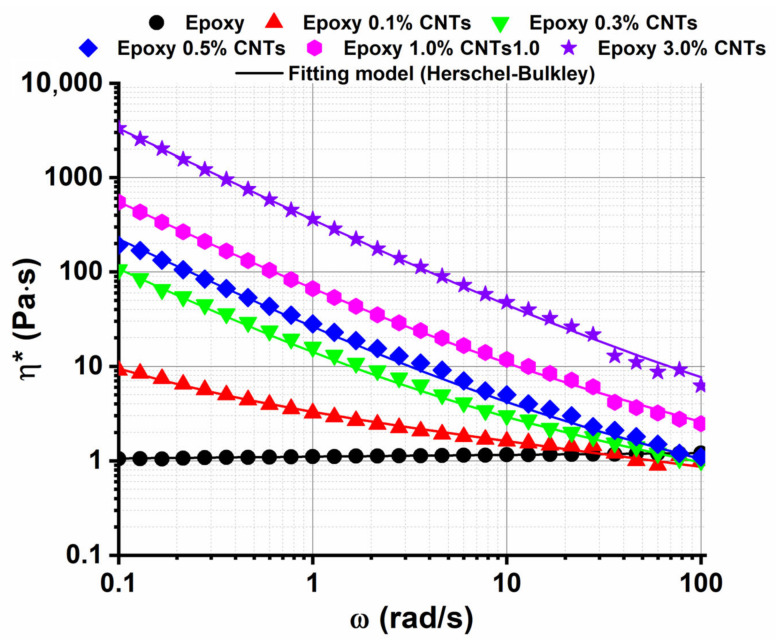
Complex viscosity vs. the angular frequency (in log–log scale). Discrete data points represent experimental data, while the lines are regression fitting of the Herschel–Bulkley model.

Figure 5 shows the evolution of the complex viscosity as a function of the frequency (in log–log scale) for epoxy resin and the nanocomposites. Epoxy resin presents a Newtonian behavior, i.e., the viscosity constant with the variation of the frequency. By increasing the filler concentration, the viscosity trend shows an evident shear-thinning behavior with respect to the Newton flow of the pristine epoxy resin in the same range of frequency. Moreover, at higher frequency values, the shear-thinning trend brings the higher viscosity values of nanocomposite materials close to the viscosity value of epoxy resin. 

When high enough shear rates are applied, the carbon nanotube network breaks down and the nanoparticles can disaggregate and/or align in the direction of the flow [62], causing a decrease in the viscosity. Usually, the change from Newtonian to shear-thinning behavior depends on the nanoparticle shape [63] and the interactions between particles and/or polymer–particles [64]. Hence it is assumed that the phenomena above described can be applied to our filled system [22]. Previous works [22,65,66,67] reported that fitting with the empirical Herschel–Bulkley model can describe the non-linear viscoelastic behavior of dispersion of the nanoparticle in a polymer. The fitting equation shown in Equation (4) represents the Herschel–Bulkley model adapted for oscillatory frequency sweeps [22,65]:

(4) $$\eta^{*}=\frac{\tau_{0}}{\omega }+k\omega^{\left(n-1\right)}$$

where η* is the complex viscosity, ω is the angular frequency, *τ*_0_ is the yield stress, *k* is the consistency index, and *n* is the flow behavior index. When *n* > 1, the fluid exhibits a shear thickening behavior, when *n* = 1 the fluid has Newtonian behavior, and when *n* < 1 the fluid shows shear-thinning behavior. 

As shown in Figure 5 and Table 2, the Herschel–Bulkley model well fits the experimental data. For the systems filled with carbon nanotubes with an amount ≥ 0.3%, the flow behavior index in the range value between 0.55 and 0.65 is indicative of a characteristic shear thinning response. For lower percentages by weight (0.1%) of CNTs, the flow behavior index still indicates shear thinning, n = 0.74), but to a lesser extent, which is the sign of the presence of a Newtonian component characteristic of the unfilled resin (n = 1). The theoretical model corroborates the tendency of the yield stress to increase with the amount of MWCNTs attributed to a denser nanofiller network. If the electrical data are compared with the rheological one, a strong correspondence in the values of the electrical and rheological percolation threshold is found, both in the concentrations range of 0.1–0.5%. The electrical and rheological data, together with the SEM investigations, show that for a concentration by weight > 0.5%, the carbon nanotube network seems to be uniformly constituted into the epoxy matrix.

### 3.2. Self-Sensing Properties

**Figure 6:**
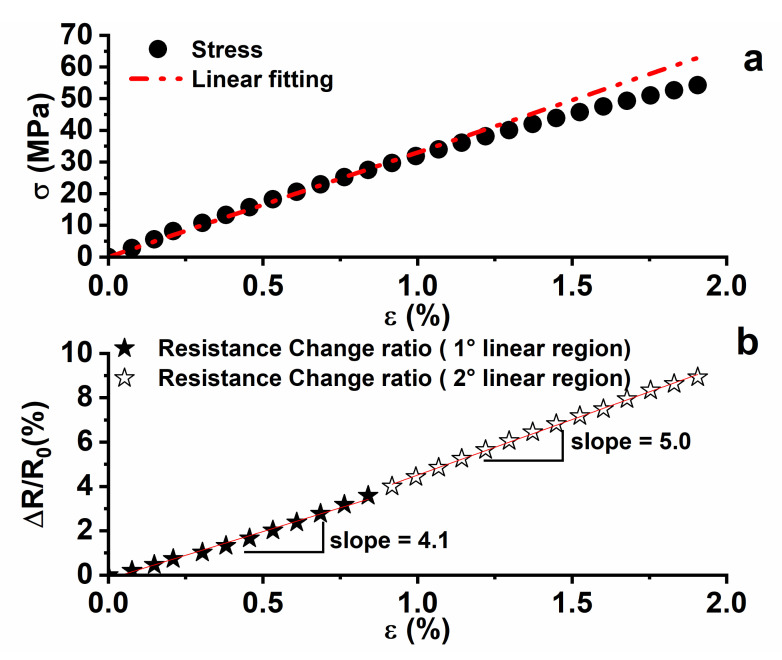
(**a**) Mechanical response and (**b**) resistance change ratio vs. the axial strain for the epoxy system filled with 0.5% by weight of the carbon nanotubes.

Figure 6a shows the mechanical response, under axial tensile stress, of the sample with 0.5 wt.% of MWCNTs measured up to the failure obtained around the strain value of ε = 2%. In more detail, a linear response is observed up to strain value around ε = 1%, for which a Young Modulus value of 3.6 GPa has been calculated. A deviation from the linear response is found for strain values higher than ε = 1%. Above this transition, plastic deformation occurs, causing permanent damage; for this reason, its value is fundamental in the structural design of the material. Therefore, for deformation lower than 1%, the material has an elastic response. The same phenomenon can be detected by considering how the percentage variation of electrical resistance (see Figure 6b) ΔR/R_0_ changes with the mechanical strain (ε). R_0_ is the electrical resistance of the sample at rest (i.e., ε = 0), and ΔR = R − R_0_ is the resistance variation when the axial load is applied. Moreover, in the region of the elastic response of the material (i.e., ε < 1%), an initial linear dependence of ΔR/R_0_ by the applied strain with a slope of 4.1 has been found. From this point on, the curve presents a linear region with an increase in the detected slope of 5. The change in the slope is probably ascribed to a permanent deformation occurring when the mechanical regime becomes plastic.

It should be remembered that the material used belongs to the family of epoxy resins with a high value of glass transition temperature, and are therefore very rigid and with almost completely elastic mechanical behavior, and for which the plastic deformation is not very evident. The epoxy system used makes it possible to evaluate the before-mentioned variations through an evaluation of the electrical resistance change of the sample. 

**Figure 7:**
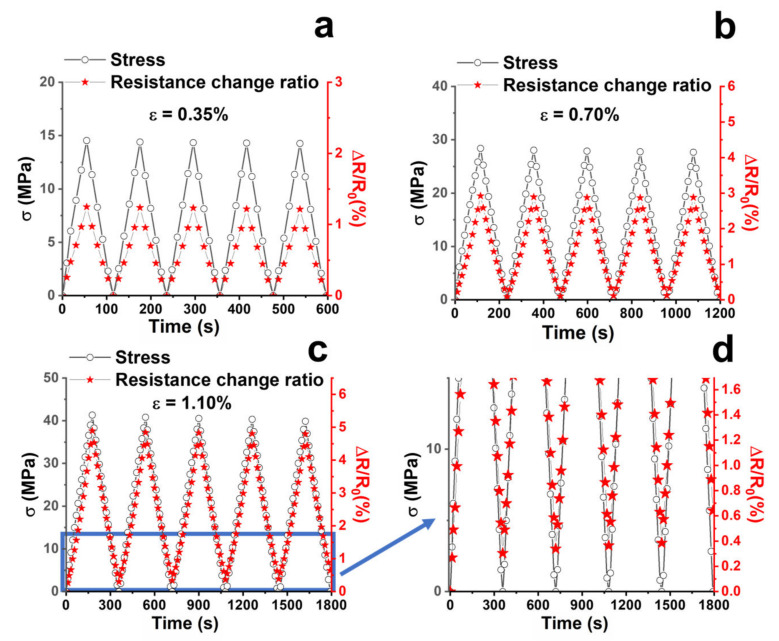
Mechanical stress (σ) and percentage resistance variation (ΔR/R_0_) over time when the applied strain is (**a**) ε = 0.35%; (**b**) ε = 0.70%; (**c**) ε = 1.1%; (**d**) enlargement of Figure 7c in the region of start conditions.

The phenomenon of the increase in the overall resistance of the sample due to the application of a load that causes an increase in the tensile strain is based on the “tunneling effect”, according to which an appreciable flow of electrons is found only if the distances among the conductive nanoparticles are sufficiently close to the so-called “tunneling distance” [68,69]. This last is affected by the CNTs concentration, the interphase thickness, surface energy, and size of CNTs. More specifically, the high levels of CNT waviness, interphase thickness, filler concentration, the fraction of networked CNT, and surface energy of CNT, as well as thin and short CNT, poor percolation threshold, and low surface energy of the polymer matrix shorten the tunneling distance [70]. In any case, the application of a load can cause both an increase in the distance between the conductive particles and/or a decrease in the contact areas between the particles, thereby changing the tunneling resistance and therefore the resistance of the whole sample. As a result of these two phenomena, the resistance of the whole sample increases. The sensitivity of the sample is expressed through the evaluation of the gauge factor expressed as G.F. = (ΔR/R_0_)/ε, which is the ratio between the variation of the normalized resistance and the axial elongation (strain). In our case, a value of 4.1 was obtained from the slope of the linear trend in the strain range < 0.7%. The second linear region was not considered in the evaluation of the gauge factor, because for strain values higher than 1% the material presents irreversible deformations. This last phenomenon is detectable in Figure 7. To obtain this graph, the material has been tested through cyclic tensile loading–unloading tests by setting the increasing value of strain (i.e., 0.35%, 0.70%, and 1.1%) and monitoring the piezoresistive response of the material.

In Figure 7a–c, for each cycle, ΔR/R_0_ always reaches the same maximum value, evidencing the repeatability of the resistive behavior of the composites. If the applied strain belongs to the elastic regime (see Figure 7a,b) for ε < 1% (i.e., ε = 0.35% and ε = 0.70%), ΔR/R_0_ values return to zero after each cycle when the load is removed (i.e., σ = 0). This means that no significant irreversible damage has occurred to the structure. By contrast, for a strain value of 1.1% (see Figure 7c), the detected residual resistance indicates the occurrence of permanent damages. In fact, a ΔR/R_0_ in the range 0.3–0.6% corresponds to ε = 1.1% (see Figure 7d, which is the enlargement of Figure 7c). This evidence can be explained as follows: when a plastic deformation happens in the material, the electrical network made of carbonaceous filler undergoes a partial rupture, causing the rearrangement of CNTs. Therefore, the microscale damages appear directly related to the resistance changes and are, hence, detectable in a non-destructive way. Indeed, the sensing material enables the identification of damage that would otherwise not be visible [46,61,71].

### 3.3. Self-Heating Properties

**Figure 8:**
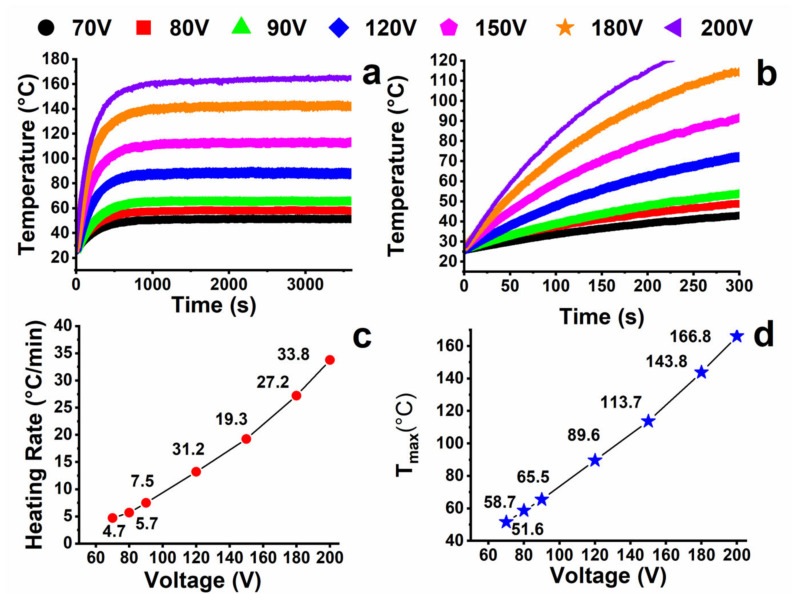
(**a**) Relationship between time and temperature at different applied voltage values at the environmental temperature; (**b**) enlargement of the first zone (time < 300 s) of Figure 7a; (**c**) heating rate at different applied voltage; (**d**) maximum temperature values at different applied voltage.

The temperature behavior vs. the heating time for different applied voltage values (from 70 V to 200 V) of the sample loaded with 3% by weight of MWCNT at room temperature is shown in Figure 8a.

The chronological trend shows that the temperature quickly increases and then reaches a maximum temperature value after a few minutes of heating. The equilibrium temperature of the sample increases as the voltage values increases and the time required for reaching the equilibrium temperature becomes longer. An enlargement of the first zone of Figure 8a, corresponding to the time range below 300 s (see Figure 8b), highlights that during the first heating times, the trend is linear and that the slope of the linear region increases with the applied voltage. In these conditions, it is possible to define a heating rate from the slope of the corresponding curves (see Figure 8c). The rate was found to be very high, from a minimum of 4.7 °C/min for a voltage value of 70 V to a maximum of 33.8 °C/min for a voltage value of 200 V. The maximum temperature value (T_max_) increases with the voltage (see Figure 8d), in an almost linear way. In any case, a variation of temperature of 40 °C can be reached in a few minutes (between 1 min and 5 min) with an appropriate applied power value. The high heating rate and achieved temperature make the epoxy composite suitable for the production of heating structural element materials, which require high thermal stability along with high temperature achieved in a short period.

**Figure 9:**
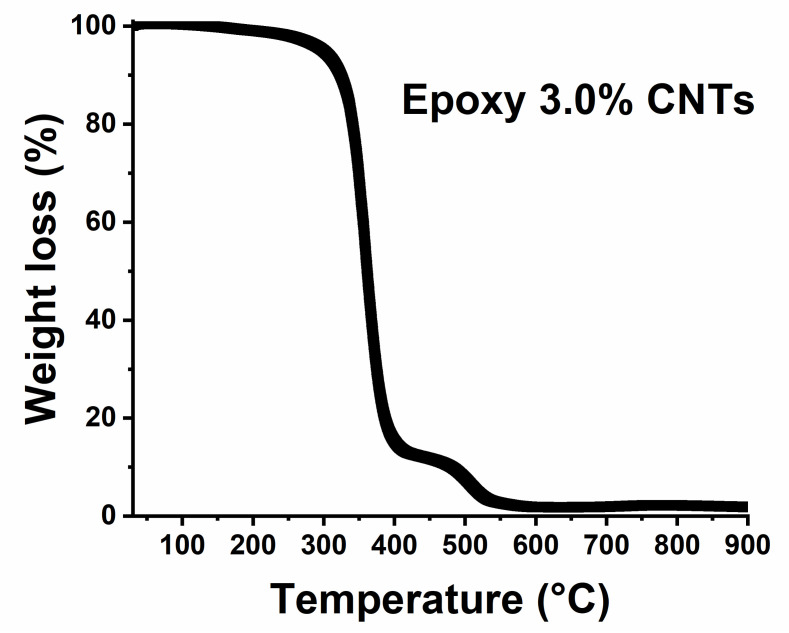
TGA curve of the epoxy formulation with the 3.0% by weight of CNTs.

The material’s thermal stability was ascertained through thermogravimetric analyses, as shown in Figure 9.

Figure 9 shows the trend of weight loss with temperature. The curve results in the main fall of about 80%, followed by a smaller step of 20%. This behavior, which presents a double degradation step, is well-known for thermosetting polymers, where the oxygen is consumed primarily by gas-phase oxidation reactions during the flaming burning of the nanocomposites; oxygen hardly reaches the thermally degrading sample surface beneath the evolved gaseous products [72]. The second stage of weight loss of the resin is referred to as the oxidation of the residual formed from thermal decomposition during the first step [73]. The onset of thermal degradation, expressed as the 5% weight loss of the sample, is around a temperature value of 297 °C, well above the temperature values reached during the electro-heating tests. 

**Figure 10:**
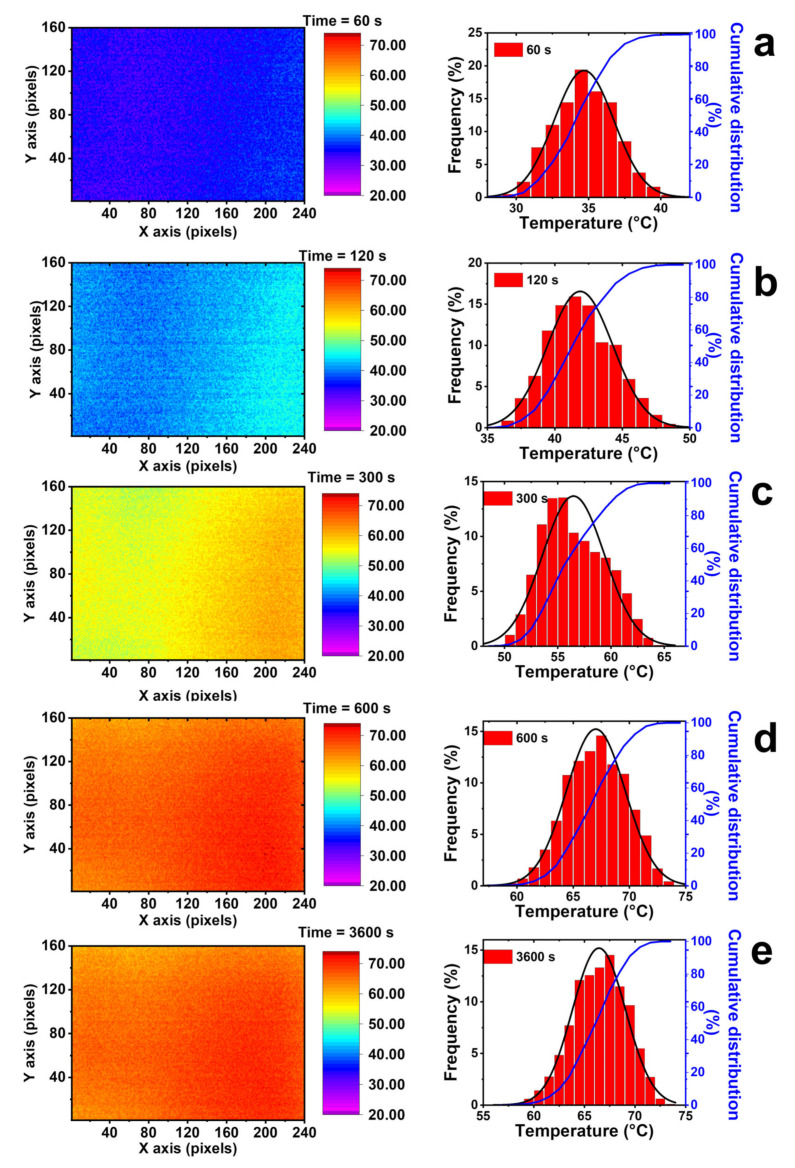
Thermal images in the central region and relating distribution temperatures of the 3% formulation, for an applied voltage of 90 V, at different heating times: (**a**) 60 s, (**b**) 120s, (**c**) 300 s, (**d**) 600s, and (**e**) 3600 s. In the temperature distribution images, the solid black lines are fitted by normal distribution curves and the solid blue lines are the cumulative distribution curves.

The detected temperature value in the center of the sample has been obtained over almost the entire surface of the sample if edge effects are neglected, as observed in Figure 10, where thermal images show the temperature mapping of the MWCNT system during heating, for an applied voltage of 90 V.

It clearly can be seen that the composite conducts heat in a very effective way by providing a homogeneous temperature map. The observed homogeneity of color tone is translated in unimodal temperature distribution, obtained for all considered times. The narrow temperature distribution allows for defining the voltage as a single technological parameter in heating management. Furthermore, the found isotropy in the heat transfer confirms the uniform distribution of the conductive filler in the epoxy matrix. The experimental data obtained during the heating tests were modeled considering the rectangular geometry shown in Figure 1 and the following Equation (5)

(5) $$C\;\frac{dT}{dt}=P-Sh\left(T-T_{ext}\right)$$

where *C* is the heat capacity, *S* is the surface of the heating element (80 mm × 55 mm), *P* is the overall heat generation due to the Joule effect (in Watt), *T_ext_* is the room temperature, and h is the total heat transfer coefficient. By applying a voltage value *V* [V] to the ends of the sample, the electrical current starts to flow inside the conductive material, characterized by an electrical resistance, *R* [Ω]. The electrical power, *P* [W], converted to generated heat according to the Joule effect is reported below:

(6) $$P=V\cdot I=\frac{V^{2}}{R}\;$$

Equation (6) is well known as Joule’s first law. Combining it with Equation (5), the following law is obtained concerning the temperature of the sample:

(7) $$T=T_{ext}+\frac{P}{Sh}\;\left(1-e^{-\frac{t}{\tau }}\right)$$

where *τ* is defined as

(8) $$\tau =\frac{C}{hA}\;$$

**Figure 11:**
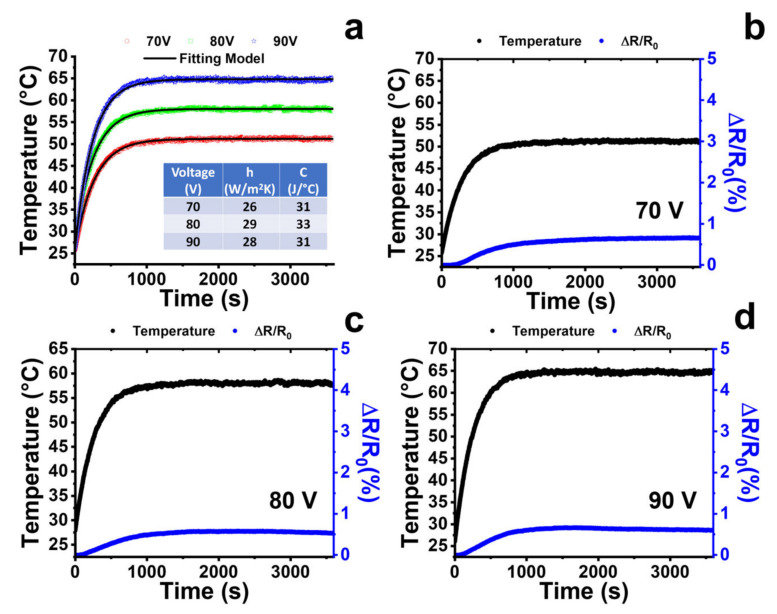
(**a**) Temperature against time during the heating test at different applied voltage values (70, 80, 90 V): symbols depict experimental data and lines depict the fitting model by Equation (7). Temperature behavior (on the left vertical axis) and resistance variation (i.e., ΔR/R_0_ on the right vertical axis) during the heating time at an applied voltage value of (**b**) 70 V; (**c**) 80 V; (**d**) 90 V.

Figure 11a shows the fitting of the experimental data obtained with Equation (7) for the heated nanocomposite, applying voltage values of 70, 80, and 90 V, respectively.

The good fitting allowed evaluation of the heat transfer coefficient (h) and the heat capacity; more specifically, a value around 27 W/m^2^ K and 32 J/°C, respectively. The results obtained with Equation (7) were possible thanks to the fact that the electrical resistance of the sample changes very little with the temperature. Considering the applied voltage values (70, 80, 90 V), the temperature values are less than 70 °C. In this range, the resistance of the sample varies by a maximum of 0.6%, as can be seen in Figure 11b–d. Furthermore, the resistance value is constant with the heating time under steady-state conditions. Thus, it is legitimate to consider the applied power constant during the heating.

**Figure 12:**
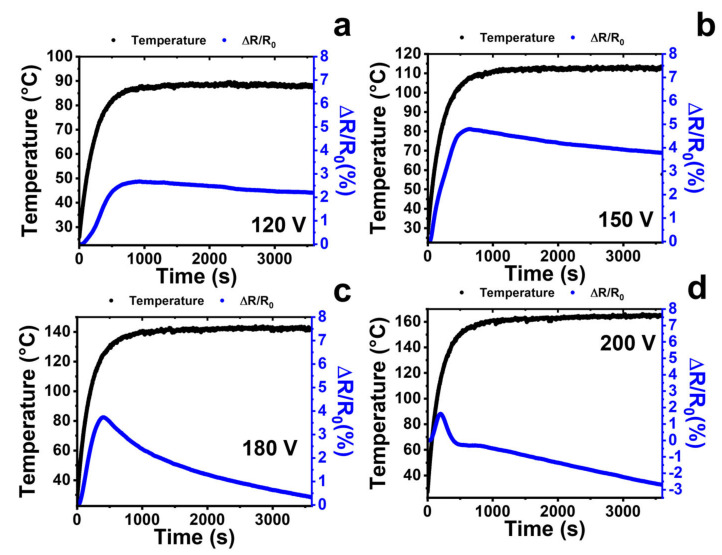
(**a**) Temperature behavior (i.e., temperature left vertical axis) and resistance variation (i.e., ΔR/R_0_, right vertical axis) vs. the heating time at an applied voltage value of (**a**) 120 V; (**b**) 150 V; (**c**) 180 V; (**d**) 200 V.

Different behavior is obtained if higher applied voltage values are applied than the previous ones, with consequent temperature values exceeding 80 °C. Figure 12 shows the trend of the resistance vs. the time for the applied voltages of 120, 150, 180, and 200 V and, simultaneously, the temperature values reached by the sample.

It is possible to observe a trend of the resistance common to all the four considered voltage values, that is an initial increase corresponding to a Positive Temperature Coefficient (PTC) trend followed by a decrease in resistance value corresponding to a Negative Temperature Coefficient (NTC) trend. The positive variation of the resistance is about 2.5% for the system heated to 120 V and then has an NTC trend when the sample achieves in steady state (see Figure 12a). The 150 V heated sample (see Figure 12b) has a higher variation of resistance up to 4.5%. It then decreases more sharply than the system heated to 120 V. When the temperature exceeds 120 °C, as in the case where a voltage of 180 V is applied (see Figure 12c), the positive change in resistance is reduced at value < 4%, and then decreases drastically to the initial value. For an applied voltage of 200 V (see Figure 12d) and temperatures of 150–160 °C, the phenomenon is repeated more clearly and the NTC behavior seems to prevail over the PTC almost immediately, reaching resistance values lower than the initial ones. 

This non-monotonous behavior can be explained if we take into account the phenomena related to the change of resistance with temperature. The impact of temperature on electrical resistance has been studied considering that the conduction mechanism under thermal activation in non-crystalline materials, like our epoxy resin, is governed by expansion strain-induced bandgap changes and hopping conductions within carbon nanoparticles [74,75]. The electrical resistance in the carbon-filled material, for concentration above the electrical threshold percolation, can be approximated as a series of resistors. Above a critical temperature range, the volume expansion phenomena occur, causing an increase in the interparticle distance. Due to the effect of an enlargement of the interparticle distance, the conduction mechanism is controlled by the tunneling effect, which leads to the common PTC effect [76]. When the interparticle distance remains unchanged, the contact resistance should decrease with increasing temperature because of the increase in the energy and mobility of the electrons, showing an NTC behavior defined as Variable Range Hopping (VRH) [76]. Other authors attribute the NTC effect to the considerable mobility of the polymer chains, with temperature values close to the matrix transitions of the polymer causing the carbon filler to stretch and close. The rearrangement of the conductive network that follows leads to the NTC effect, generating more electrical contact points [76,77]. 

**Figure 13:**
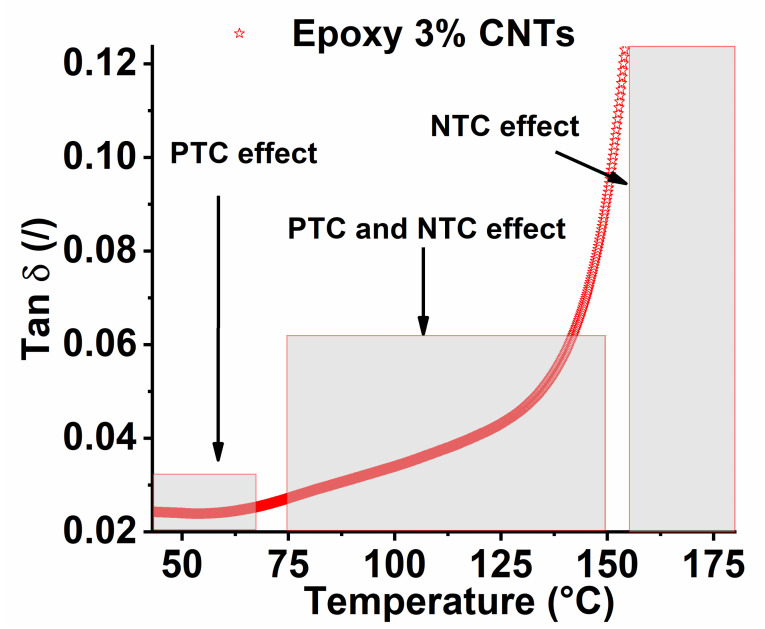
Loss factor (tan δ) vs. the temperature in the region near the glass transition temperature.

The described phenomenon so far can be explained if we consider Figure 13, which shows the trend of the loss factor (Tan δ) vs. the temperature obtained from DMA analysis.

It is important to note that near the glass transition region, the loss factor is controlled by free volume available within the polymer, then by the chain segment mobility [78]. An external input, such as heat, favors the conformational movements of chain segments, causing a variation of the free volume. These features lead us to conclude that the shape of the Tan δ curve can be interpreted as a result of a relaxation phenomenon occurring at various temperatures [53]. In more detail, relaxation phenomena influence the arrangement of the conductive network, made up of carbon nanotubes, and can explain the resistance variations observed during the heating process.

Many of the assertions above refer to thermoplastic polymers that have a glass transition temperature below the investigation range of temperature. In our case, the resin has an amorphous structure with a glass transition temperature of around 200 °C (see Figure 13a). Below the glass transition temperature, the chain segments exhibit poor mobility, which does not significantly increase if the temperature is below 75–80 °C. In this condition, the system exhibits a PTC effect due to the expansion phenomena of the free volume of the matrix. For temperature values above 80 °C, the effects of the proximity of the glass transition temperature begin to manifest themselves, determining the appearance of the NTC phenomenon. The more the heating temperature approaches the glass transition temperature, the more the NTC effect tends to prevail over the PTC phenomenon. For example, as shown in Figure 12d for temperature values of 160 °C, for which the system is in full glass transition (see Figure 13), the behavior is almost completely NTC.

## 4. Conclusions

In this paper, the role of MWCNT loading has been intensively investigated. Depending on the intelligent functionalities required, it is possible to design a nanocomposite material on the basis of filler concentration by moving along the profile of the electrical percolation curve. Once a compatible matrix-filler system is defined, the potentialities of this high-impact approach allow for obtaining cutting-edge structural self-sensing or self-heating elements. In the present work, a high-performing epoxy resin has been produced with different MWCNT contents. AC and DC characterization of the obtained materials have been conducted to determine the electrical parameters and the percolation threshold (around 0.5%). The morphological and rheological analyses have proved the homogeneous distribution of the filler into the matrix. The self-sensing properties, analyzed for the 0.5% MWCNT formulation, display a G.F. (4.1) significantly higher than the commercial extensometer (around 2) and allow avoiding the adjunct of an external sensing element, because the bulk material is the sensor itself. Moreover, the self-heating tests have been performed with the same nanocomposite system, but at higher filler content (3%), to operate at lower applied voltage. On the one hand, the presented self-heating device can heat up to high temperatures in a very short time, giving it the possibility to be applied for different applications, from aeronautics to automotive. 

Nevertheless, the resistance profile has evidenced how more phenomena simultaneously occur during the heating tests. The PTC behavior is dominant below 80 °C because the electrical resistance increases mainly for thermal expansion of the system, which leads to an increase in the distances between the conductive nanoparticles. By contrast, the first relaxation phenomena occur above 80 °C, as evidenced by the DMA analysis. 

For this reason, during the self-heating tests at higher steady-temperature, a non-monotonous trend of the resistance is recorded. The relaxation phenomena lead reasonably to higher mobility of the polymer chains that favors the current flow. For this reason, above 80 °C, both PTC and NTC phenomena are reported, and the NTC phenomenon is dominant in full glass transition above 150 °C.

## Figures and Tables

**Table 1:** Preparation procedure sample and characterization methods.

Material/Method	Supplier/Device	Specific	Procedure Measurement/ Technical Specifications
Precursor: 3,4Epoxycyclohexylmethyl-3’,4’-epoxycyclohexane carboxylate” (ECC) Hardener: Methyl hexahydrophthalic anhydride” (MHHPA); Carbon nanotubes (GRAPHISTRENGTH C100)	Precursor/hardener: Gurit Holding Wattwil, Swiss; Carbon nanotube:s: ARKEMA Colombes, France; Hielscher model UP200S-24 kHz high power ultrasonic probe: Hielscher Ultrasonics, Teltow, Germany.	The manufacturing procedure of the samples: According to Ref. [43]	Curing cycle: 1 h at 80 °C + 20 min at 120 °C +1 h at 180 °C.
Scanning Electron Microscopy (SEM)	JSM-6700F, (JEOL Akishima, Japan)	-	Etching procedure: According to Ref. [47]
The electro-mechanical characterization: Dinanometer; strain gauge; electrical measurements	INSTRON, series 5967-INSTRON, Norwood, MA, USA; (RS 632-180, RS PRO, Corby, UK);Multimeter 3458A (Agilent, Santa Clara, CA, USA)	Tensile test: crosshead speed of 1 mm/min	According to Refs. [46,47]; Sample production: ASTM D638 standards [45]
Electrical conductivity measurement DC/AC	Electrometer: Keithley 6517A (Keithley Instruments, Cleveland, OH, USA); Quadtec7600 dielectric analyzer (IET Labs Inc., Roslyn Heights, NY, USA)	Two-probe method; AC frequency range [102,103,104,105,106]	According to Refs. [44,46]
Dynamic mechanical analysis (DMA)	TA instrument-DMA 2980, USA	(35 × 10 × 4mm^3^)	Mode: Dual Cantilever 1 Hz −60 to 260 °C 3 °C/min^−1^
Thermogravimetric analysis (TGA)	Mettler TGA/SDTA 851 (Mettler-Toledo, Columbus, OH, USA)	Range temperature: 30–900 °C	Heating rate 10 °C min^−1^.
Rheological Measurement	Haake Mars II (Thermoscientific, Waltham, MA, USA) rotational rheometer	Frequency Range 0.1–100 rad/s T = 25 °C	According to Ref. [29]

**Table 2:** Herschel–Bulkley model parameters.

Sample	τ_o_ (Pa)	K (Pa∙s^n^)	N (/)	R^2^
Epoxy	0	1.11	1.02	0.980
Epoxy 0.1% CNTs	0.42	2.88	0.74	0.996
Epoxy 0.3% CNTs	9.81	4.34	0.65	0.998
Epoxy 0.5% CNTs	21.00	5.27	0.60	0.986
Epoxy 1.0% CNTs	50.94	15.82	0.56	1.000
Epoxy 3.0% CNTs	322.62	35.26	0.55	1.000

## Data Availability

Not applicable.

## Supplementary Materials

The following supporting information can be downloaded at: https://www.mdpi.com/article/10.3390/nano13030495/s1, Figure S1: Infrared spectra of the components of the resin: (a) precursor, (b) hardener; Figure S2: Infrared spectra of the CNTs; Figure S3: (a) C1s and (b) O1s scans of the powders of CNTs. (c) Elemental composition of the CNTs determined by XPS, (d) Percentage of groups of CNTs determined by XPS; Figure S4: Raman spectrum of CNTs; Table S1: Assignment of Raman bands of CNTs to the different vibration modes.
