# Supplementary material for: Role of MWCNTs Loading in Designing Self-Sensing and Self-Heating Structural Elements
Source: Nanomaterials (Basel). 2023 Jan 26;13(3):495. doi: 10.3390/nano13030495 (PMC9920374; doi:10.3390/nano13030495)
Supplement: Supplementary file 1 [file nanomaterials-13-00495-s001.zip › nanomaterials-2160260-supplementary.pdf]

nanomaterials

MDPI

Supplementary Materials

Role of MWCNTs loading in designing self-sensing and self-heating structural elements

Liberata Guadagno [1], Raffaele Longo [1], Francesca Aliberti [1], Patrizia Lamberti [2], Vincenzo Tucci [2], Roberto Pantani [1], Giovanni Spinelli [3,4], Michelina Catauro [5] and Luigi Vertuccio [5,*]

[1] Department of Industrial Engineering, University of Salerno, Via Giovanni Paolo II, 132, 84084 Fisciano, Italy; lguadagno@unisa.it (L.G.); rlongo@unisa.it (R.L.); faliberti@unisa.it (F.A.); rpantani@unisa.it (R.P.)

[2] Department of Information and Electrical Engineering and Applied Mathematics University of Salerno, Via Giovanni Paolo II,132, 84084 Fisciano, Italy; plamberti@unisa.it (P.L.); vtucci@unisa.it (V.T.)

[3] Faculty of Transport Sciences and Technologies University of Study "Giustino Fortunato", Viale Raffaele Delcogliano 12, 82100 Benevento, Italy; g.spinelli1@unifortunato.eu (G.S.)

[4] Open Laboratory on Experimental Micro and Nano Mechanics, Institute of Mechanics, Bulgarian Academy of Sciences, Acad. Georgi Bonchev Street., Block 4, 1113 Sofia, Bulgaria

[5] Department of Engineering, University of Campania "Luigi Vanvitelli", Via Roma 29, 81031 Aversa, Italy; michelina.catauro@unicampania.it

* Correspondence: luigi.vertuccio@unicampania.it

Citation: Guadagno, L.; Longo, R.; Aliberti, F.; Lamberti, P.; Tucci, V.; Pantani, R.; Spinelli, G.; Catauro, M.; Vertuccio, L. Role of MWCNTs Loading in Designing Self-Sensing and Self-Heating Structural Elements. *Nanomaterials* **2023**, *13*, 495. https://doi.org/10.3390/nano13030495

Academic Editors: David Jenkins; Montserrat Gomez

Received: 29 December 2022
Revised: 15 January 2023
Accepted: 25 January 2023
Published: 26 January 2023

Copyright: © 2023 by the authors. Licensee MDPI, Basel, Switzerland. This article is an open access article distributed under the terms and conditions of the Creative Commons Attribution (CC BY) license (https://creativecommons.org/licenses/by/4.0/).

In the compounds used in this study, the precursor was based on the "3,4-Epoxycyclohexylmethyl-3',4'-epoxycyclohexane carboxylate" (ECC), whereas the hardener agent was based on the "Methyl hexahydrophthalic anhydride" (MHHPA).
A Bruker Vertex 70 FTIR spectrometer equipped with an FTIR device with temperature control (Golden Gate heated single-reflection diamond ATR, Specac-Teknokroma) was used to determine the FTIR spectra of the components of the resin. The FTIR was used at a resolution of 4 cm^{-1} and 10 scans were averaged for each spectrum. Figure S1 shows the IR spectra of the precursor (Figure S1a) and hardener (Figure S1b).

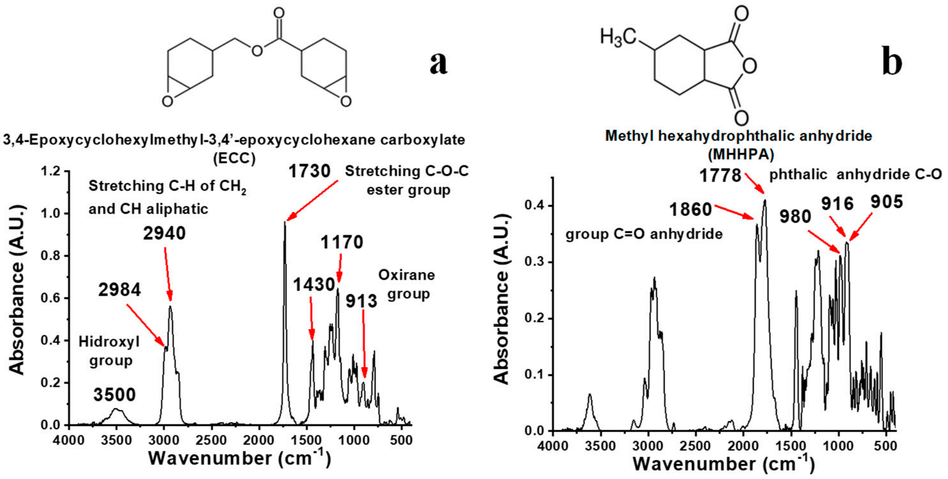

Figure S1. Infrared spectra of the components of the resin: (a) precursor, (b) hardener.

According to the literature, the wavelength of 915 cm^{-1} is the stretching of the epoxy group; the wavelength of 1730 cm^{-1} is the C=O stretching of ester group; the wavelength of 2940 cm^{-1} is CH and CH_2 of the C–H stretching situated on the aliphatic ring; the wavelength of 3000–3600 cm^{-1} is due to the O–H stretching. The IR spectrum confirms that the precursor is a specie based on the 3,4-Epoxycyclohexylmethyl-3',4'-epoxycyclohexane carboxylate (ECC). According to the literature, as can be seen from Figure 1, the peak of

the absorbance bands at 1860, 1780 and 915 cm^{-1} correspond to the C=O and C-O stretches for the cyclic phthalic anhydrides. The IR spectrum confirms that the hardener is a species based on the Methyl hexahydrophthalic anhydride (MHHPA).

The carbon purity of the CNTs is >90% with a weight loss at 105 °C <1% (value obtained by thermogravimetric analysis (TGA)). Carbon nanotubes are characterized by an outer mean diameter ranging from 10 to 15 nm, a length ranging from 0.1 to 10 µm and a number of walls varying between 5 and 15 [29].

FTIR spectroscopy has been used widely in the structural determination of functional groups bonded to carbon based nanofillers. In this work, FTIR analysis has been performed in the range 400 to 4000 cm^{-1} for the identification of the functional groups attached on the surface of CNTs.

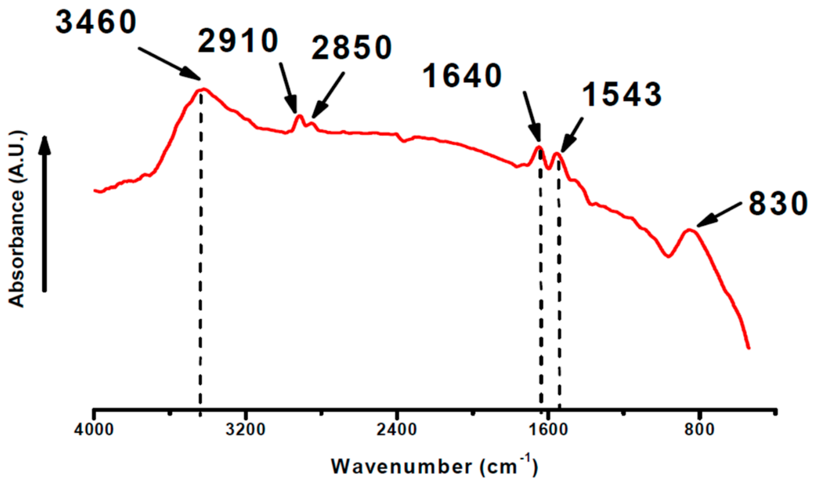

Figure S2. Infrared spectra of the CNTs.

The FTIR spectrum of CNTs (see Figure S2) shows a stronger intensification of the peaks at 3460 cm^{-1}, 1640 cm^{-1} and 1543 cm^{-1} and additional peaks at 2910 cm^{-1} and 2850 cm^{-1} corresponding to C-H stretching of oxidized multi walled carbon nanotubes, together with the peak at 830 cm^{-1} associated with first order vibrational mode of graphitic structure. The profile of the peak at 830 cm^{-1} also highlights the presence (probably on the terminations of MWCNTs) of 1-4 substitution of aromatic rings. Such results highlight that the CNTs have a great number of functional groups on the nanostructure surface. This is an ideal condition to obtain a good dispersion in epoxy matrices, which are characterized by high number of polar groups.

The elemental composition of CNTs has been determined by using X-Ray Spectroscopy XPS. All spectra were collected using Al–K_ radiation (1486.6 eV), monochromatized by a twin crystal monochromator, yielding a focused X-ray spot (elliptical in shape with a major axis length of 400 µm) at 3 mA × 12 kV. The alpha hemispherical analyzer was operated in the constant energy mode with survey scan pass energies of 200 eV to measure the whole energy band and 50 eV in a narrow scan to selectively measure the elements. XPS data were analyzed with Avantage software. A smart background function was used to approximate the experimental backgrounds and surface elemental composition were calculated from background-subtracted peak areas.

The polar groups highlighted in the FTIR analysis, are testified also by XPS measurements, where a little percentage of oxygen has been found, which determines C/O ratio of 74.76 (see Figure S3).

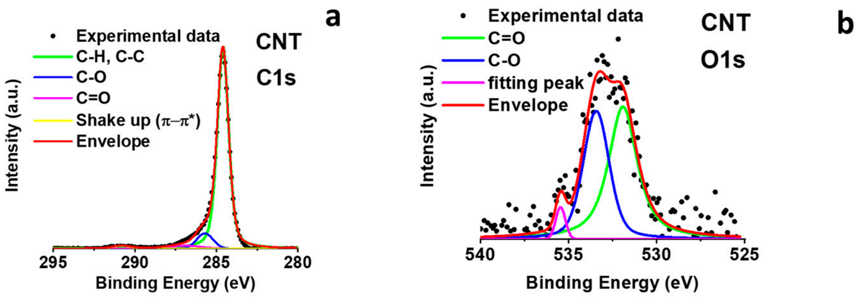

c

Element	CNTs
C [%]	98.68
O [%]	1.32
C/O	75.76

d

Bonds	Binding Energy (eV)	CNTs
C=C	284.50	97.91
C–OH/C-Csp3	285.70	7.78
C=O	287.00	2.69
Shake up (p-p*)	290.60	1.60

Figure S3. a) C1s and b) O1s scans of the powders of CNTs. c) Elemental composition of the CNTs determined by XPS, d) Percentage of groups of CNTs determined by XPS.

The CNTs sample shows the presence of a peak at binding energy (BE) of 290.1 (290.6) eV in the spectrum of C1s scan (see Figure S3), which is attributed to a shake-up satellite characteristic of the aromatic carbon, π–π* (HOMO–LUMO) transition.

Raman spectra were obtained at room temperature with a micro-Raman spectrometer Renishaw inVia with a 514 nm excitation wavelength (laser power 30 mW) in the range (400–3000 cm^{-1}).

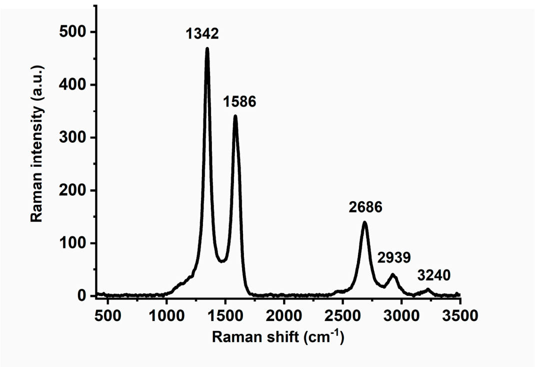

Figure S4. Raman spectrum of CNTs.

Raman spectroscopy has been used for the characterization of CNTs powder, showing typical bands of carbon nanotubes (see Figure S4). Main bands obtained in the Raman spectrum appeared at 1342, 1586, 2686, 2939 and 3240 cm^{-1}, which are ascribed to different vibration modes, as included in Table S1.

Table S1. Assignment of Raman bands of CNTs to the different vibration modes.

Raman shift (cm^{-1})	Band
1342	D band (sp3, slightly disordered graphite wall)
1586	G band (E2g mode of graphite wall, sp2)
2686	2D band
2939	D+G bands (second order)
3240	2D′ band

The Raman spectrum obtained for the CNTs powder is typical of carbon nanotubes materials. The presence of the D and G-bands in the raw CNTs suggests the presence amorphous and ordered graphitic carbon, due to the following main peaks: D peak round 1350 cm^{-1}, G peak round 1580 cm^{-1} and 2D band around 2700 cm^{-1} (overtone band) (see figure 4). The G peak is characteristic of the ordered graphitic layers and corresponds to the tangential vibration of the carbon atoms, whilst the intensity of the D band is due to the presence of defects (amorphous carbon, the higher number of defects, the higher D band intensity). The ratio of the intensity of D and G bands (I_D/I_G) is a typical parameter for carbon-based materials characterization, and it gives a measurement of the purity and quality of the carbon nanotubes [79]. In our case the I_D/I_G ratio value of 1.38 has been calculated.
